# Camera-Based Respiration Monitoring of Unconstrained Rodents

**DOI:** 10.3390/ani13121901

**Published:** 2023-06-07

**Authors:** Lukas Breuer, Lucas Mösch, Janosch Kunczik, Verena Buchecker, Heidrun Potschka, Michael Czaplik, Carina Barbosa Pereira

**Affiliations:** 1 Department of Anesthesiology, Faculty of Medicine, RWTH Aachen University, Pauwelsstraße 30, 52074 Aachen, Germany; 2 Institute of Pharmacology, Toxicology, and Pharmacy, Ludwig-Maximilians-University of Munich, Königinstraße 16, 80539 München, Germany

**Keywords:** respiration, automatic monitoring, rodent, rat, animal welfare, refinement, 3R, laboratory animals, camera-based monitoring, breathing

## Abstract

**Simple Summary:**

Monitoring vitals sign such as the respiratory rate, heart rate, or temperature is of high importance to medical and biological research. Using camera-based methods, we monitored the respiratory rate of unconstrained laboratory rats by analyzing the visible breathing movement in the thorax. We hope this is a further step to enabling the non-invasive monitoring of rodent in an experimental environment without using implanted sensors, reducing the stress and pain within an otherwise unneeded operation.

**Abstract:**

Animal research has always been crucial for various medical and scientific breakthroughs, providing information on disease mechanisms, genetic predisposition to diseases, and pharmacological treatment. However, the use of animals in medical research is a source of great controversy and ongoing debate in modern science. To ensure a high level of bioethics, new guidelines have been adopted by the EU, implementing the 3R principles to replace animal testing wherever possible, reduce the number of animals per experiment, and refine procedures to minimize stress and pain. Supporting these guidelines, this article proposes an improved approach for unobtrusive, continuous, and automated monitoring of the respiratory rate of laboratory rats. It uses the cyclical expansion and contraction of the rats’ thorax/abdominal region to determine this physiological parameter. In contrast to previous work, the focus is on unconstrained animals, which requires the algorithms to be especially robust to motion artifacts. To test the feasibility of the proposed approach, video material of multiple rats was recorded and evaluated. High agreement was obtained between RGB imaging and the reference method (respiratory rate derived from electrocardiography), which was reflected in a relative error of 5.46%. The current work shows that camera-based technologies are promising and relevant alternatives for monitoring the respiratory rate of unconstrained rats, contributing to the development of new alternatives for a continuous and objective assessment of animal welfare, and hereby guiding the way to modern and bioethical research.

## 1. Introduction

Animal research has played a major role in many scientific breakthroughs for centuries, even though it has been a source of various ethical debates [1]. This caused governing bodies to implement laws and other regulatory means to safeguard animals in experimental settings. The European Union (EU) requires member states by its Directive 2010/63/EU [2] to apply the 3R principles proposed by Russell et al. [3] in 1959. These principles refer to reduction, refinement and replacement as a mean to minimize the use of animals in scientific studies, while maximizing animal welfare. The term reduction refers to reducing the number of animals used in a study, while still providing the scientific significance needed. Refinement refers to minimizing the pain, suffering, or distress introduced by animal trials. This can be achieved by using less invasive methods or improving the living conditions in terms of housing and care. Replacement refers to finding alternatives to animal testing which are similar or more effective, thus making the animal trial needless. Feasible alternatives could be using cell cultures, simulations, or human studies.

However, reality shows that not all experiments with living animals can be replaced. In 2019, the EU reported that 10.61 million animals were still used in animal trials [4], showing the great need for further refinement methods. Of these, 72% were used for research, 17% to satisfy regulatory requirements and another 6% for routine production. Most of the animals were used to enhance the understanding of the nervous system or finding treatments for diseases such as cancer. Until today, research has not been able to find adequate replacements for these kinds of animal testing, which makes the refinement and improvement of these experiments crucial.

Due to their high anatomical, physiological, and genetic similarity to humans, while being small and easy to maintain, mice and other rodents are most used in research [5] and represent about half of all trial animals [4]. Cardiovascular, pharmacological, and toxicological research requires vital parameters such as the heart rate (HR) or respiratory rate (RR) to assess a given theory. Currently, implanted radio transponders are the only methods to monitor these for unrestrained mice or rats [6]. This can be ECG sensors, piezoelectric sensors, implanted catheter, or other implanted devices. Despite its ability to generate highly precise data, there are several significant drawbacks associated with this methodology. First, it requires an initial implantation surgery, which is invasive and time-consuming. The recovery time for animals to regain their normal circadian rhythms can take up to five to seven days, according to Braga and Burmeister [7]. Second, the implanted device may cause distress and discomfort, especially in small species. Braga and Burmeister also noted that the implanted device could have adverse physiological effects, such as an increased volume in abdominal viscera, which can potentially compromise the movement of the diaphragm and alter breathing patterns in terms of depth and rhythm. Therefore, there is a great need for contactless and unobtrusive monitoring of techniques, which, on the one hand, permit continuously monitoring the laboratory animals and on the other hand obtaining objective parameters for welfare assessment.

There are numerous examples of the application of RR monitoring for rodents, including toxicity studies in drug development [8], anesthesia monitoring [9], respiratory disease research [10], stress and pain assessment [11], sleep research [12] and many more. Ohtani et al. [8] compared the analgesic and respiratory effects of norbuprenorphine (NBN) and buprenorphine (BN), finding that BN had a lower concentration for an analgesic effect without inducing respiratory depression compared to NBN. Tsukamoto et al. [9] studied the effect of multiple anesthetics on vital signs such as temperature, heart rate, respiratory rate and SP0_2_. Card et al. [10] identified the differences in respiratory physiology depending on the sex, using a seral model of respiratory diseases. Schöner et al. [11] found out that an increased respiratory rate might occur in a model of PTSD in rats. Mendelson et al. [12] investigated sleep apnea in rats.

Over the years, numerous researchers have explored monitoring RR remotely. In 2019, Kunczik et al. [13] showed that the monitoring of mice and rats can be achieved using an RGB camera while undergoing anesthesia. In this approach, RR is measured by tracking the movement of the abdominal areas, while HR is measured using a DistancePPG, as proposed by Kumar et al. [14]. Another approach was presented by Takahashi et al. [15], using the camera recordings of mice from below a see-through acrylic glass, monitoring and tracking hairless areas. Both approaches lack the possibility of long-term monitoring as we would like to see, due to the animals being restrained or in a specialized cage with no possibility of litter or enrichment materials such as nesting pads.

The current paper presents an improved approach for respiratory rate monitoring in rodents by using visual imaging from above. In contrast to other publications that use videos of anaesthetized animals to estimate this vital parameter, our focus here is to demonstrate the capability of the presented algorithm in extracting this from moving animals.

## 2. Materials and Methods

**Figure 1:**
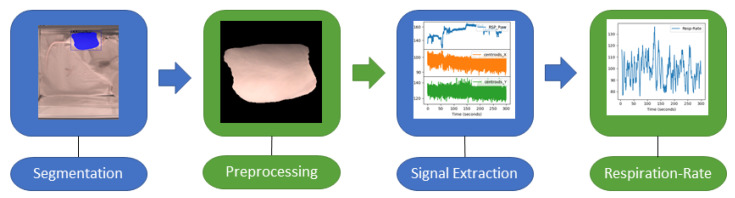
Key stages involved in extracting the RR from the RGB videos of rats: Video preprocessing (segmentation, preprocessing), signal extraction, and RR calculation.

The proposed algorithm is a multi-step approach for monitoring respiration in an RGB video of unconstrained rats, as illustrated in Figure 1. This paragraph provides a brief overview of all steps, which will be described in detail in the following sections, along with the experimental protocol. During the first step, segmentation masks of images are computed from video recordings using a deep learning algorithm to detect the respiration-associated movement. In the second step, the preprocessing of the segmented regions is carried out. In the third step, the signal is extracted. Last, the actual computation of the respiratory rate is carried out. As a reference, respiration signals were extracted from electrocardiography (ECG) data and used to compare the camera-based signal.

### 2.1. Experimental Protocol

The data used in this work are part of a larger study that adhered to the 3R principles (replacement, refinement and reduction) to ensure the ethical treatment of animals. The study followed the approved experimental protocol of the governmental animal care and used the institution “Regierung von Oberbayern” (Germany, ROB-55.2-2532.Vet_02-16-105), and was conducted in compliance with the German Animal Welfare Law. All animals received humane care in accordance with the principles outlined in the “Guide for the Care and Use of Laboratory Animals” (8th edition, NIH Publication, 2011, USA).

**Figure 2:**
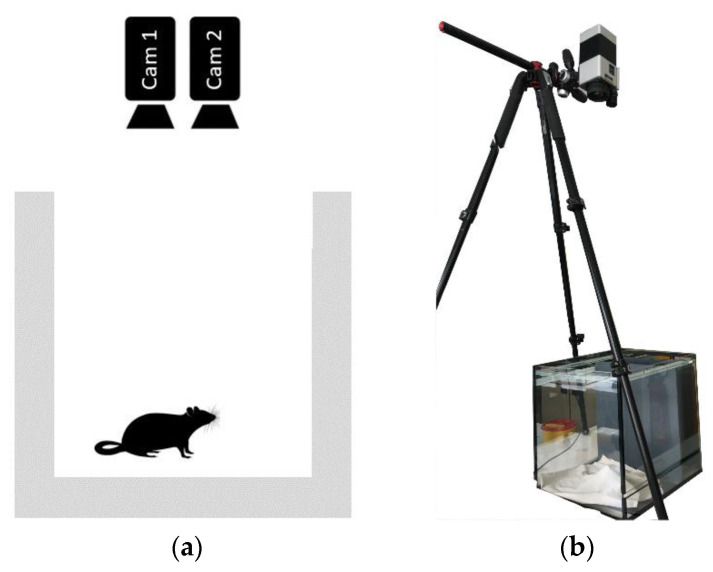
Recording setup. (**a**) Schematic view with both the RGB and thermal camera, which are recording the rat from above. (**b**) Picture of the recording setup. Both cameras were mounted using a tripod 1.5 m above the monitoring cage.

Three male albino Sprague Dawley rats (360–375 g; 9–11 weeks; Envigo, Horst, The Netherlands) were included in this study. They were subjected to an operation in which ECG and EEG transponders (DSI-HDX02, Data Sciences International, Inc., New Brighton, MN, USA) were implanted. A detailed description about the surgical procedure was already published in 2019 by Seiffert et al. [16]. Prior to and following the operation, the rats were placed into an open glass cage, measuring approximately 0.30 m × 0.30 m, and recorded using two cameras (Cam1 and Cam2). The cage was bedded with a white textile sheet and no additional illumination was provided. The cameras were mounted above the cage on a tripod at about 1.5 m above the bottom of the cage. The distance was selected so that both cameras could acquire the complete bottom of the cage. The experimental setup is depicted in Figure 2.

Cam1 is a long-wave infrared thermal camera (Infratec VarioCAM HD head 820, InfraTec GmbH, Dresden, Germany) with a resolution of 640 × 480 pixels, a thermal resolution of up to 20 mK, a frame rate of 60 FPS and a dynamic range of 16 bit. Cam2 is an RGB camera (Allied Vision Mako G-223C, Allied Vision Technologies GmbH, Stadtrova, Germany) with a resolution of 1368 × 640 pixels and a framerate of 60 FPS, resulting in 18,000 images for a 5 min recording per modality.

**Figure 3:**
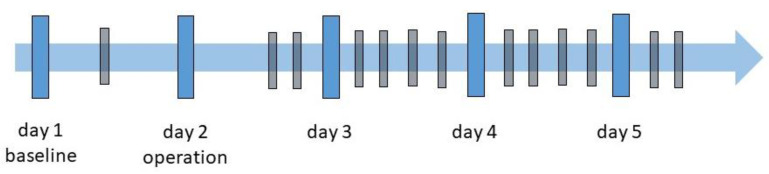
Experiment schedule: The blue bars correspond to the five measurement days. The black bars indicate the times at which the recordings were made.

The experiment was conducted over five consecutive days, as shown in the experiment schedule displayed in Figure 3. At each measurement time (MT), two 5 min videos were recorded with a parallel ECG recording:

- Day 1: One video recording was obtained for establishing a baseline and the rats allowed to acclimate to the environment. For this recording, no ECG was recorded.
- Day 2: This was surgery day where the EEG and ECG transponders were implanted. Two recordings with all three rats were carried out: the first directly after the surgical procedure and the second approximately two hours later.
- Days 3 to 5 followed a similar schedule, with recordings starting at 9 am, 11 am, 1 pm and 3 pm. On day 5, only the first two video acquisitions were made.

For every recording, the ECG transponder had to be activated using a magnetic switch. Shortly afterward, the camera recordings were started simultaneously for both cameras. After 5 min of recording time, the cameras switched off automatically, followed by activating the magnetic switch again to turn off the transponder. This allowed for the recording of 13 videos for each rat, 5 min each, totaling to 39 videos (in total 195 min of video recordings). All videos were captured in raw format, without any compression. During the recording, the rats were allowed to move freely, resulting in occasional sections of heavy movements, while most of the videos are made up of minor movements such as sniffing, and fur care is present in all of the videos.

After the experiment, the animals were euthanized with an intraperitoneal sodium pentobarbital injection (600 mg/kg Narcoren^®^, Merial GmbH, Hallbergmoos, Germany).

### 2.2. Segmentation

For assessing the heart rate, a target RoI must be defined. In contrast to previous works, which mostly monitored anesthetized animals using on the upper abdomen as the region for signal extraction [8], our goal was to monitor unconstrained animals. This means that the RoI must be detected and tracked over time. Thus, the RoI was set to cover the entire chest and abdomen, and was bounded by the connecting line between both upper and lower legs, which can be recorded by cameras when they are mounted above the cage.

**Figure 4:**
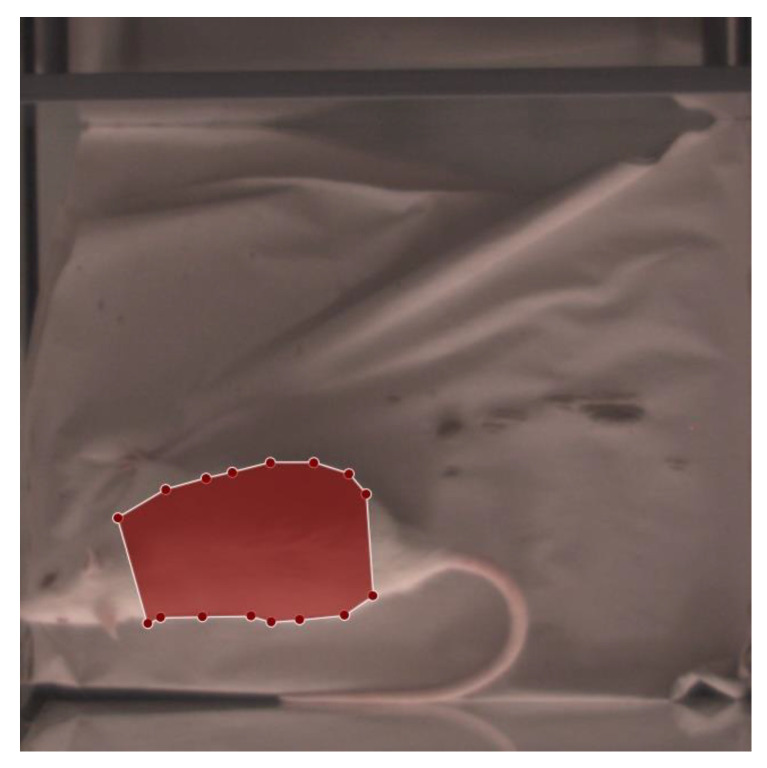
Annotated rat images: The red area corresponds to the desired RoI (thorax and abdomen), which should be automatically identified and segmented.

In 2019, Wu et al. [17] published the detectron2 framework for image segmentation and object detection, which was customized for segmenting the RoI in rats in this work. Such supervised deep learning approaches need annotated image data before the training process of the neural network can be started. Therefore, images from our study (described in detail in Section 2.1) were selected, such that 50 images that were automatically extracted from each of the 39 recorded videos, beginning with images with little-to-no movement and then randomly sampling until the required number (50) was reached. These images were annotated using LabelMe, a project created by the MIT Computer Science and Artificial Intelligence Laboratory (Cambridge, MA, USA), which provides an annotation tool to build image databases for computer vision research. An example of an annotation can be seen in Figure 4, which was applied in RGB images. Along with the detectron2 framework, Wu et al. [17] also published pretrained models on various datasets. To begin training our network, the Mask-RCNN-R50-FPN architecture, was chosen, which was pretrained on the CoCo-Dataset [18] (referenced as model-ID: 137849600). Mask-R-CNN-R50-FPN references a deep learning model, for instance segmentation. As a backbone, a ResNet-50 is used, consisting of 50 convolutional layers to extract the features from the input image. These features are then used in a feature pyramid network (FPN) to build a multi-scale feature pyramid for improved object detection and segmentation.

To adapt Mask-R-CNN R50 FPN to the current data, minor changes were made to its architecture. Appendix A provides a complete set of the changed parameters of the model architecture. The feature extraction layers of the network were frozen, and the number of RoI-heads was set to 128 to enable a batch size of 8 during training. Training was performed using a GeForce RTX 2080 Super (NVIDIA Corporation, Santa Clara, CA, USA). To evaluate the neural network properly, the dataset was divided into three parts (training, validation, test), with each part containing data from a single rat. For each rat, a network was trained on the 650 annotated images per rat, validated on a second rat, and tested on a third rat. This is done to ensure that the neural network had not been exposed to any images of the animals included in the test data, and thus prevent any bias during the evaluation caused by any animal-specific visible features. During training, several augmentations were applied (see Appendix B for a complete set of augmentations). Applying the segmentation network to each frame of the video results in two different outputs: a binary mask, and a certainty score between 0 and 1. Detections which are exceeding a score 0.99 were defined as valid segmentations.

### 2.3. Preprocessing and Signal Extraction

For an RR assessment from the segmented images, several steps of preprocessing were performed. Based upon the binary masks, from the segmentation step, the centers of the mass were computed, and each image was cropped to the extent of the bounding box of the segmentation mask, after nullifying every pixel outside the segmented area. Subsequent to obtaining all masked images of a given video, the images were shifted so that the centers of mass are overlapping for each frame in a video. The preliminary respiration signal R was obtained by computing the area of the segmentation in each image. To extract the signal, R was denoised using a linear denoising algorithm according to Nowara et al. [19], which was originally developed for denoising remote photoplethysmography signals, but should be also applicable for respiration signals due to a similar temporal profile.

The noise signals include the linear detrended center-of-mass coordinates over time for both X- and Y-coordinates, as well as their first derivatives. The algorithm uses the disturbed signal R projected onto the noise subspace *Q* to compute the denoised signal *Z* with $Z=R-\frac{QQ^{T}}{Q^{T}Q}R$. Furthermore, the resulting signal was preprocessed with a second-order Butterworth bandpass filter, with a lower and upper cutoff frequency of 1 Hz (60 breaths/min) and 3.3 Hz (200 breaths/min), respectively, and clipped wherever the gradient exceeded 1.5. The clipped values were then filled by interpolating the two neighboring values of the respiration signal.

### 2.4. RR Computation

**Figure 5:**
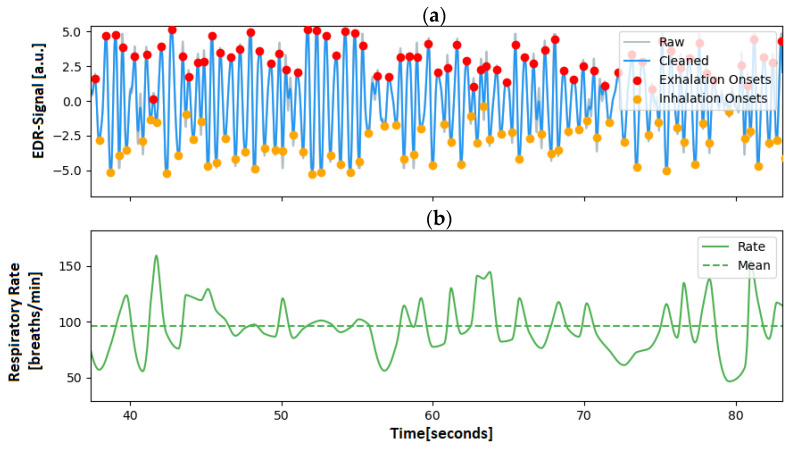
Example of ECG-derived respiration signal and the rate extracted from a rat ECG; (**a**) EDR signal: The blue line corresponds to the EDR signal, on which the red dots represent the maximum and the yellow dots the minimum of the breathing signal. (**b**) The EDR rate is the corresponding instantaneous respiratory rate, with its mean value denoted as a dashed line.

Once the filtered respiration signal has been acquired, a peak detection is carried out to determine both in- and exhale cycles, which can later be used to compute the RR. An algorithm developed for electrical impedance tomography (EIT) by Khodadad et al. [20] was adapted for this purpose. First, the signal was detrended by subtracting the means of a best-fit line, and zero crossings in the signal were found. Second, a separate search for extreme points at both rising and falling zero crossings was performed. Third, an outlier detection algorithm was applied to identify the valid peaks based on their distance from the neighboring peaks. Once the peaks have been computed, the instantaneous RR (f_RR_) can be calculated as the inverse of the distance between two consecutive peaks, using the equation: f_RR_ = 60/d_peak_, where d_peak_ corresponds to the number of sampling points divided by the sampling rate and the respiration signal f_RR_ is given in breaths per minute (breaths/min). Figure 5 illustrates the algorithm, showing two signals an ECG-derived-respiration signal at the top and the corresponding computed RR at the bottom.

### 2.5. ECG Analysis and ECG-Derived Respiration

The results were validated using ECG as the ground truth, since the radio transponder employed in the animal trial allowed for the extraction of this parameter. ECG-derived respiration (EDR) describes the process of extracting the respiration signal from a given ECG signal. However, to obtain an EDR signal of interest, the processing of the raw ECG signal was required.

**Figure 6:**
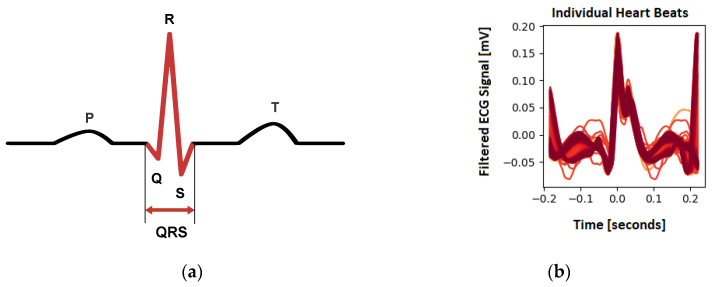
Heartbeat in ECG signals; (**a**) Schematic diagram of an ECG of a human. (**b**) Showcase of individual heart beats by ECG of the captured rats in the experiment.

Several methods were proposed for peak detection in an ECG signal, including by Pan et al. [21], Vuong et al. [22], Kalidas et al. [23], Koka et al. [24] and Makowski et al. [25]. Most of these methods focus on detecting the QRS complexes of a given ECG as it is the most prominent feature. The peak detection method used was proposed by Makowski et al. [25], who used the gradients’ steepness to detect QRS complexes, followed by searching the local maxima within the detected region to find the R-peak. Customization was required to enable the computation of the HR of rats, as their ECGs have a morphology that is vastly different from that of humans. The schematic ECG of a normal human is shown in Figure 6, along with the recorded an ECG of a rat.

**Figure 7:**
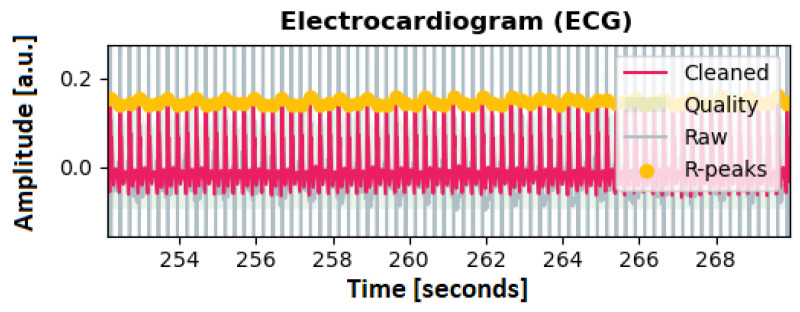
ECG signal of a rat, including the utilized peak detection, as denoted by the yellow markers.

The customization involves filtering the signal with a Butterworth low-pass filter, with a cutoff at 4 Hz, and discarding possible artifacts resulting from a 50 Hz powerline frequency. To apply the peak detection method to rats, the kernel size for smoothing and averaging was reduced by factors of two and four (smoothwindow = 0.05 s; avgwindow = 0.1875 s), respectively. Additionally, the minimum delay between two different peaks was set to 0.1 s. The threshold for discarding a QRS complex because it is too short was set to 0.1 s. An exemplary detection of the resulting R-peaks can be seen in Figure 7.

**Figure 8:**
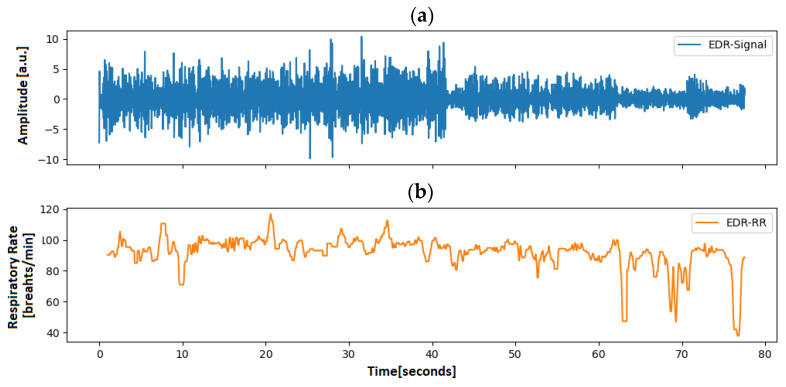
ECG-derived respiration in rats. (**a**) ECG-derived respiratory waveform after applying the approach proposed by van Gent et al. [22]. (**b**) Respiratory rate of the animal computed according to Khodadad et al. [23].

**Figure 9:**
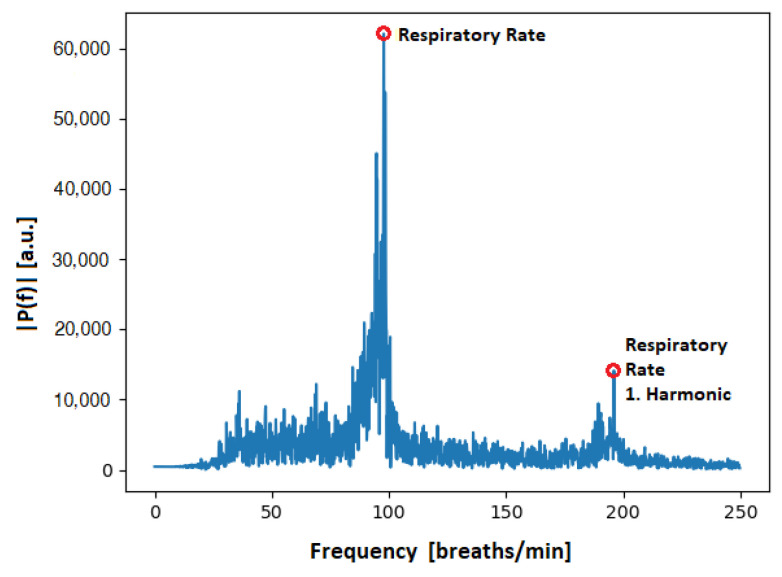
Frequency spectrum of a rat’s respiratory signal. The highest peak visible at around 100 breaths/min corresponds to the respiratory rate of the animal. Additionally noticeable is the first harmonic, around approximately 200 breaths/min.

Many methods have been proposed to extract the EDR from an ECG signal. Sarkar et al. [26], Charlton et al. [27] and van Gent et al. [28] used simple filtering to reconstruct the respiratory signal, while Kontaxis et al. [29] computed the respiratory signal from the difference between the maximum and the minimum slopes in the QRS complex. Langley et al. [30], in turn, computed the EDR signal by applying principal component analysis of the global amplitude variation of the QRS complex. To receive the respiratory signal from our data, the approach from van Gent et al. [28] was used, as it was most robust, especially when used on noisy signals. An EDR signal computed with this method can be seen in Figure 8, along with its respiratory rate. Figure 9, in turn, shows the spectrum of a processed ECG spectrum, clearly showing the respiratory rate and the first harmonic.

## 3. Results

### 3.1. Reference Respiratory Rate

**Figure 10:**
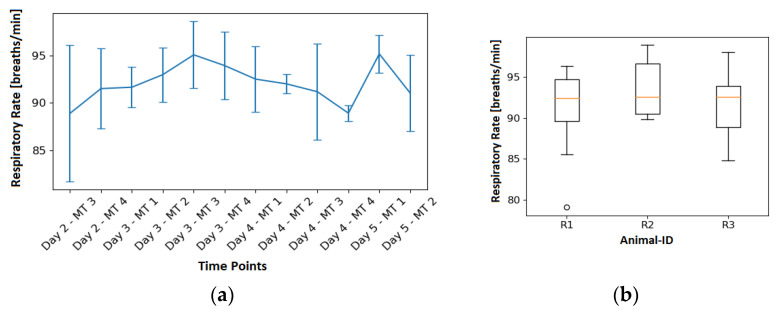
EDR results: (**a**) Illustration of the temporal aspect of the RR by grouping measurements for the boxplot by measurement time. (**b**) Boxplot of all measurements split by the different animals.

Figure 10 shows the RR derived from the ECG for each measurement time point, as well as a box plot diagram showing the variation of the ECG-derived RR for each animal. Looking at the results, it can be observed that the RR ranges from 79.08 breaths/min to 98.87 breaths/min. On average, 92.09 breaths/min was recorded, with a standard deviation of 4.23 breaths/min. A detailed list of respiratory rates for all measurement time points is reported in Table 1.

### 3.2. Segmentation

**Figure 11:**
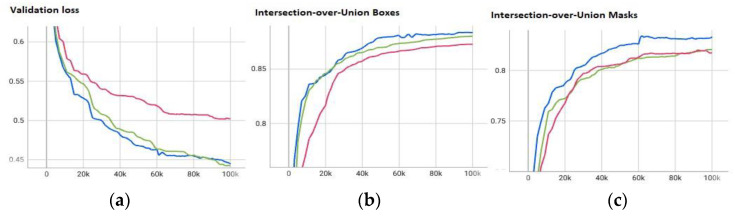
Validation loss (**a**) and intersection-over-union (**b**,**c**) for the trained networks. Blue: Network trained on R1, validated on R2, tested on R3. Green: Network trained on R2, validated on R1, tested on R2. Pink: Network trained on R3, validated on R1, tested on R2.

The neural networks were trained on the images of one rat each, over the time of 100,000 iterations, thus leaving the images of the other two rats for validation and testing. Throughout the training process, the weights of the neural network were saved periodically every 10,000 steps and validated on the validation set, as is shown in Figure 11. The figure is split into three parts, showing the validation losses, intersection over union (IoU) for the detected bounding boxes and the IoU of the segmentation masks for each of the three trained networks over time. At the end of the training process, the network-weights with the smallest validation loss were selected for the evaluation of the test set.

Intersection over union is defined as the area of overlap divided by the area of union IoU = A_intersection_/A_union_. Overall, the segmentation on the test data resulted in an average IoU of 87.75% ± 5.04% for the segmentation masks, and an IoU of 82.52% ± 6.69% for the bounding boxes. Even though the networks were trained on different animals, only small differences can be seen in the IoU scores. Table 2 shows the detailed results for the two IoUs, along with the subjective certainty score computed by the network for all three rats, along with the average.

### 3.3. Respiratory Rate

**Figure 12:**
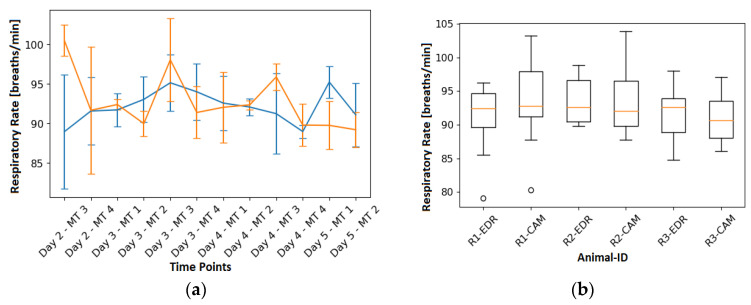
EDR ref vs. camera-based RR: (**a**) RR over time for each MT and its variation as a boxplot. EDR rate is shown in blue, while the orange curve is the camera-based RR. (**b**) Boxplot of all results grouped by animal and modularity. R1-EDR is the EDR rate of R1 and R1-CAM is the camera-based RR for R1.

In the left part of Figure 12, the EDR (blue) can be seen together with the RR computed from the RGB videos (orange) for each measurement time point. In turn, the right part is showing the variation of the EDR and camera-based RR for each animal. In addition, Table 1 shows the RR for each video that was analyzed and an average RR of the reference. As can be observed in the table, the relative error averaged 5.47%, while the absolute error was 4.95 breaths/min.

## 4. Discussion

The aim of this research paper is to assess the feasibility, and accuracy of monitoring RR in unrestrained, awake laboratory rats using visible imaging. This is of particular interest considering that previous approaches have only been performed with sedated animals, which does not correspond to reality for most respiratory monitoring applications. Drug development and toxicity studies could especially benefit from the possibility of long-term respiration monitoring, allowing for the assessment of the side-effects with only little interaction with the animal care takeover, while also minimizing the cost and labor for telemetry implants and evaluation. Furthermore, recovery after transmitter implantation would not be needed. Anesthesia monitoring could also profit from the proposed methods, even though it is not of much benefit to replace the current monitoring devices during an operation, as automatic respiration monitoring could be used to ensure a safe recovery from anesthesia without the need of care-takers to be present. For most respiratory diseases, it is necessary to monitor the actual breaths rather than the respiratory rate. Since the outliers are removed to enable monitoring with movement present in the signal, our methods might not be suitable for signal extraction when they are later used for the classification of complex breathing patterns. Stress and pain assessment might be one of the most interesting perspectives for this method, since pain and stress assessment is becoming more and more important in animal experiments.

The results confirm the successful performance of the segmentation and tracking algorithm; it accurately identified the thorax and abdominal area as the RoI and effectively tracked them, achieving an IoU of the segmentation mask of 87.74% on average. Unfortunately, due to the absence of enrichments in the open glass cage, image occlusion testing could not be carried out. However, based on the inherent nature of the algorithm, we have strong confidence in its ability to perform effectively, even when the animal is occluded and reappears in the image. The respiratory waveforms were extracted by leveraging the cyclical changes in the size of the area of the RoI caused by the expansion and contraction of the thorax during the respiratory cycle. Despite the presence of challenging conditions, such as motion artifacts caused by the animal’s movement in the cage, the RR could still be extracted with a high degree of accuracy from the videos, with the absolute error averaging 4.95 breaths/min, providing a fist proof of the concept which has to be validated further in future studies with more animals. Nevertheless, the error could be further minimized by reducing the overall coverage. In this work, all available video sequences were used for RR estimation and evaluation. Therefore, animal movement leads to movement artifacts and thus higher errors between the reference and RR computed from visual imaging. Additionally, an ECG-derived RR rate is not the most accurate ground truth as it is very prone to motion artifacts. Other sensors, such as an implanted subcutaneous piezoelectric, may provide a more accurate reference. Varon et al. [31] also reported that EDR is quite prone to errors from noisy ECG signals. This is caused by faulty peak detection propagating into the respiration signal. Nonetheless, alternative gold-standard methods, such as respiratory belt transducers, require the animal to be restrained during the RR measurements.

There are other studies in the literature that aimed to extract the respiratory waveform/RR from rats noninvasively. Wang et al. [32] and Guan et al. [33] used humidity sensors to evaluate the RR of rodents, but both methods require the animal to be restrained. These studies primarily focus on describing the sensors themselves and the extracted respiratory waveform, but lack comprehensive investigations and comparisons with a reference/ground truth. Esquivelzeta Rabell et al. [34] and Kurnikova et al. [35] used camera-based methods to monitor respiration, namely thermal and visual imaging. In these studies, the focus was not on the RR itself, but rather the waveform of the respiratory curve extracted from the temperature variation around the nostrils, to analyze exploratory sniffing. As a result, the parameter RR was not calculated further. The algorithms used required a close-up view of the animal’s nostrils, with minimal motion involved. In 2019, Kunczik et al. [13] extracted the RR from six anesthetized laboratory rats. The results have demonstrated excellent algorithm performance, with a root-mean-square error of 0.32 breaths/min. It is worth highlighting that the animals were under anesthesia during the study, and thus the influence of motion artifacts on the algorithm performance was not tested. In a study by Anishchenko et al. [36], the RR of laboratory rats during sleep was remotely measured using a radar, webcam and thermal camera, yet no reference for validation purposes was acquired, which makes a direct comparison with the present approach unfeasible.

While the tests in this study were conducted on rats, the algorithm developed can potentially be applied to other rodents such as mice and hamsters, though retraining the tracking algorithm would be necessary, along with minor adjustments, such as modifying the parameters of the temporal filter to adapt to the expected RR range of the specific animal species.

In relation to the presented study, there are some limitations that should be discussed as they may have influenced the results. First, the similar colors of animals and background (both white) might have impaired the algorithm and most probably decreased the overall accuracy, as the contrast between both is very low. Moreover, when considering the approach for denoising the respiration signal, it solely focuses on the general relative movements and does not consider movements such as scratching or sniffing during the denoising process, which could potentially affect the accuracy of the results. Inaccuracies of the tracking might have also contributed to more noise in the respiration signal, and thus a smaller signal-to-noise ratio. To further enhance the results, a dynamic assessment of the exposure time setting for the camera depending on the illumination of the RoI could be beneficial. This assessment would involve adjusting the exposure time based on the RoI rather than the overall lighting environment. By tailoring the exposure time to the specific RoI, more accurate and precise measurements could be obtained, leading to improved outcomes. Another possibility to improve the results, and thus the overall accuracy, would be to decrease the coverage of the algorithm by considering only those videos sequences in the extraction where no movement is present. However, this would imply that continuous monitoring would no longer be possible. In this context, the question arises as to whether continuous monitoring is really indispensable in laboratory research or whether fewer measurements, for example one measurement per hour, would be sufficient. Obtaining a short video sequence (e.g., 10–20 s) of motionless animals could potentially be adequate for this purpose. This could potentially minimize the monitoring burden, while still providing sufficient data for analysis, depending on the specific research objectives and requirements. Further investigation and validation would be necessary to determine the optimal frequency and duration of measurements for the specific research context. Another potential limitation is that the current segmentation algorithm is not real-time capable, but this could be improved with a different architecture. If continuous monitoring is not necessary, then the algorithm does not necessarily need to be real-time capable.

Overall, the proposed algorithm can evaluate the RR of unconstrained rodents properly. Further studies will focus on the application of the developed methods in a home cage scenario, to assess the feasibility of continuous long-term monitoring and the robustness over a wider range of respiratory rates.

## 5. Conclusions

Until today, it was not possible to replace animal research entirely in medical and biological science. Therefore, the need for the further refinement of experiments is significant. Vitals signs, such as the respiratory rate, are mostly monitored by using ECG implants. Until now, camera-based methods only allowed for monitoring the respiratory rate in anesthetized animals, thus a new method was proposed for unconstrained and moving animals. The respiratory rate was analyzed through the cyclical expansion and contraction of the rats’ thorax/abdominal region. Compared to the EDR, a relative error of 5.47% could be achieved, while the IoU of the segmentation mask of the thorax region averaged 87.74%.

Improvements and further experiments are still needed to evaluate the performance of the algorithm when animals are occluded; furthermore, a higher range of respiratory rates is needed to evaluate the robustness of this approach. This could enable a fully automatic camera-based monitoring of rodents, reducing the need for implanted transmitters and thereby surgeries in animal experiments.

## Figures and Tables

**Table 1:** RR from camera-based respiration compared to the EDR. For each day and time of the measurement, the table shows the EDR rate, the camera-based RR (Rrcam), as average over the whole measurement. Additionally, the resulting relative error and the absolute error are listed. The last row lists the average of all recorded values.

Day	MT	Rat ID	Mean EDR [Breaths/min]	Mean RR_cam_ [Breaths/min]	Rel. Error [%]	Abs. Error [Breaths/min]
Day 2	MT3	R1	96.28	99.56	3.41	3.28
		R2	79.08	98.63	24.72	19.55
		R3	91.34	103.23	13.02	11.89
	MT4	R1	94.05	80.29	14.63	13.76
		R2	85.55	97.73	14.24	12.18
		R3	94.97	96.83	1.96	1.86
Day 3	MT1	R1	94.61	92.88	1.83	1.73
		R2	90.69	82.72	8.79	7.97
		R3	89.70	91.45	1.95	1.75
	MT2	R1	96.28	91.64	4.82	4.64
		R2	93.45	90.37	3.30	3.08
		R3	89.27	87.77	1.68	1.5
	MT3	R1	98.73	99,01	0.28	0.28
		R2	96.32	103.9	7.87	7.58
		R3	90.27	91.15	0.97	0.88
	MT4	R1	98.87	89.18	9.80	9.69
		R2	92.41	88.86	3.84	3.55
		R3	90.60	95.97	5.93	5.37
Day 4	MT1	R1	97.40	90.03	7.57	7.37
		R2	89.8	87.75	2.33	2.09
		R3	90.34	98.22	8.72	7.88
	MT2	R1	92.79	92.75	0.04	0.04
		R2	92.74	91.49	1.35	1.25
		R3	90.55	92.68	2.35	2.13
	MT3	R1	97.29	97.04	0.26	0.25
		R2	84.8	93.48	10.24	8.68
		R3	91.48	97.03	6.07	5.55
	MT4	R1	89.24	88.32	1.03	0.92
		R2	89.74	87.41	2.60	2.33
		R3	87.79	93.49	6.49	5.7
Day 5	MT1	R1	98.03	93.67	4.45	4.36
		R2	93.69	86.25	7.94	7.44
		R3	93.89	98.2	4.59	4.31
	MT2	R1	93.86	91.41	2.61	2.45
		R2	85.3	86.09	0.93	0.79
		R3	93.95	89.95	4.26	4
	Ø		92.09	92.67	5.47	4.94

**Table 2:** IoU segmentation algorithm: The table shows the results for all three trained networks, Rat ID denotes the rat on which the evaluation was performed. N describes the number of images which were annotated for the corresponding rat and used for testing. IoU is the percentage of the intersection of both annotated and detected RoIs, once computed with the rectangle (IoU Box) around the RoI, and once with the pixelwise-mask of the segmented area (IoU-Mask). The certainty score is the computed certainty that a rat was found in the segmented area.

Rat ID	N	IoU Box [%]	IoU Mask [%]	Certainty Score [%]
R1	637	82.27 ± 7.73	86.86 ± 6.18	99.84 ± 0.40
R2	654	82.85 ± 6.01	88.28 ± 4.61	99.90 ± 0.26
R3	659	82.42 ± 6.37	88.09 ± 4.39	99.80 ± 1.69
Ø	650	82.52 ± 6.69	87.75 ± 5.04	99.85 ± 0.79

## Data Availability

The data presented in this study are available on request from the corresponding author. The data are not publicly available due to the file size of the raw videos.

## Appendix A

**Table A1:** Network parameters. Table of parameters, changed from default parameters in the Detectron 2 RGB model.

Parameter	Value	Description
INPUT.MIN SIZE TRAIN	(480, 512, 544, 576, 608, 640)	Size of short edge for rescale
INPUT.MIN SIZE TEST	(480)	Size of short edge for rescale
SOLVER.IMS PER BATCH	8	Batch size
SOLVER.BASE LR	0.0001	Learning rate
SOLVER.MAX ITER	100,000	Number of training iterations
MODEL.ROI HEADS.BATCH SIZE PER IMAGE	128	Number of regions of interest heads
MODEL.SEM SEG HEAD.LOSS WEIGHT	2	Weight for segmentation loss
MODEL.ROI HEADS.NUM CLASSES	1	Number of classes

## Appendix B

**Table A2:** Image augmentations. Table of applied image augmentations in Detectron 2.

	Augmentation
1.	RandomBrightness (intensity min = 0.5, intensity max = 2)
2.	RandomContrast (intensity min = 0.5, intensity max = 2)
3.	RandomSaturation (intensity min = 0.5, intensity max = 2)
4.	RandomFlip (prob = 0.5)
5.	RandomFlip (prob = 0.5, horizontal=False, vertical=True)
6.	RandomExtent (scale range = (0.8, 1.2), shift range = (0.05, 0.05))
7.	RandomRotation (expand = False, angle = [−15, 15], interp = BILINEAR)
8.	ResizeShortestEdge (short edge length = INPUT.MIN SIZE TRAIN, sample style = “choice”, max size = 1368)
